# Supplementary material for: DMRforPairs: identifying Differentially Methylated Regions between unique samples using array based methylation profiles
Source: BMC Bioinformatics. 2014 May 15;15:141. doi: 10.1186/1471-2105-15-141 (PMC4046028; doi:10.1186/1471-2105-15-141)
Supplement: Additional file 2 — DMRforPairs output for the comparison of A431-MCF7 and NA17018-NA17105. Please start from the HTML files in each folder. Available via the BMC Bioinformatics website. [file 1471-2105-15-141-S2.zip › 1394847754114233_MOESM2_ESM/A431_MCF7/figures/19931.pdf]

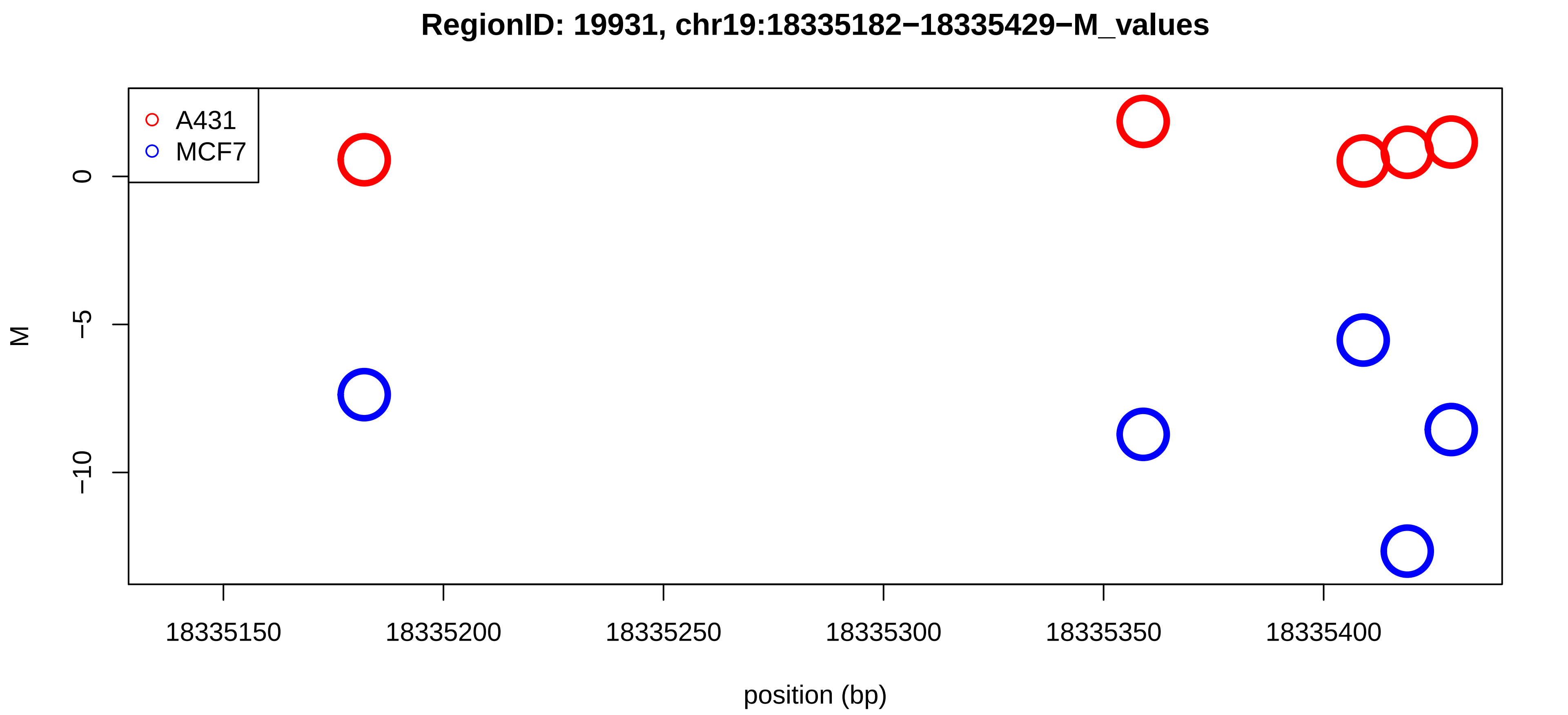
RegionID: 19931, chr19:18335182−18335429−M_values
A431
MCF7
M
0
−5
−10
18335150
18335200
18335250
18335300
18335350
18335400
position (bp)

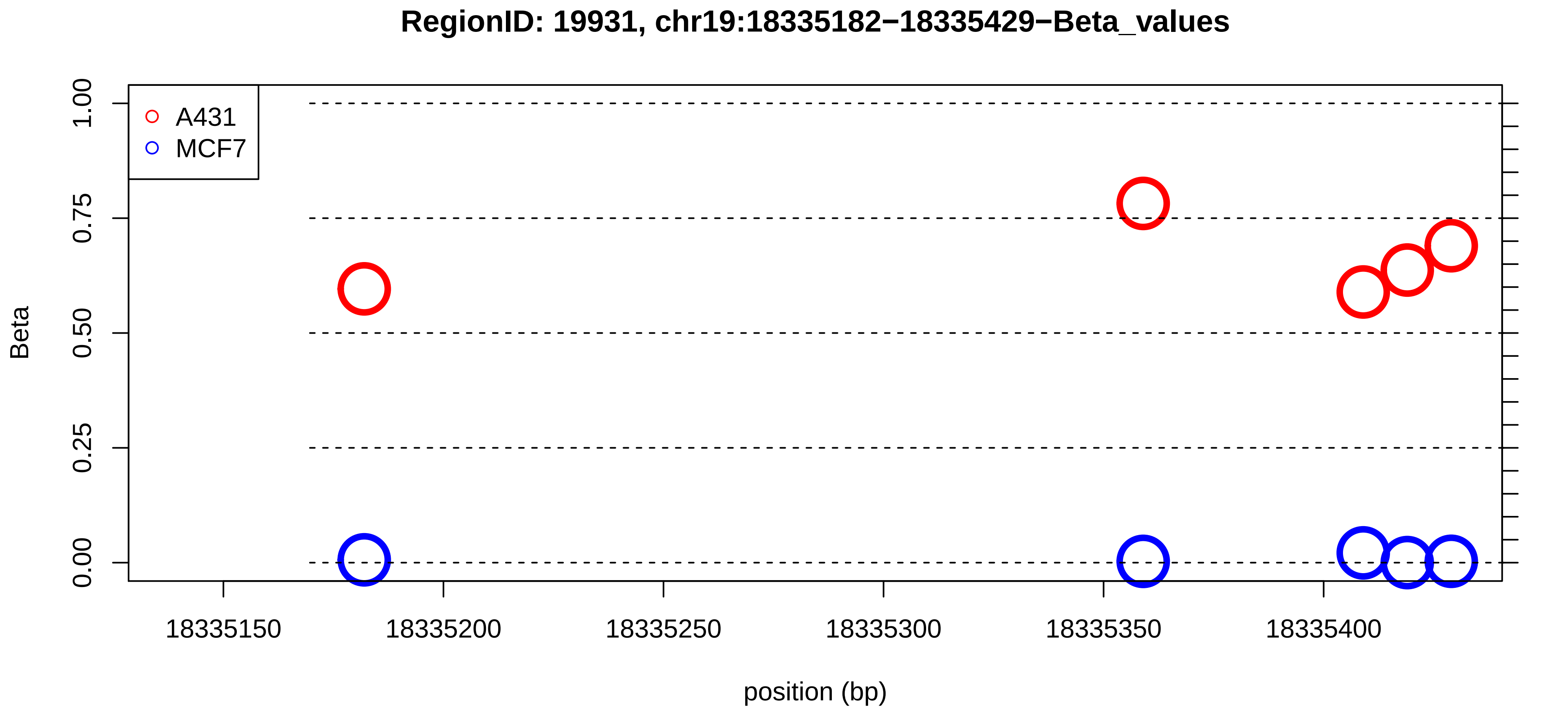
RegionID: 19931, chr19:18335182−18335429−Beta_values
A431
MCF7
Beta
0.00
0.25
0.50
0.75
1.00
18335150
18335200
18335250
18335300
18335350
18335400
position (bp)

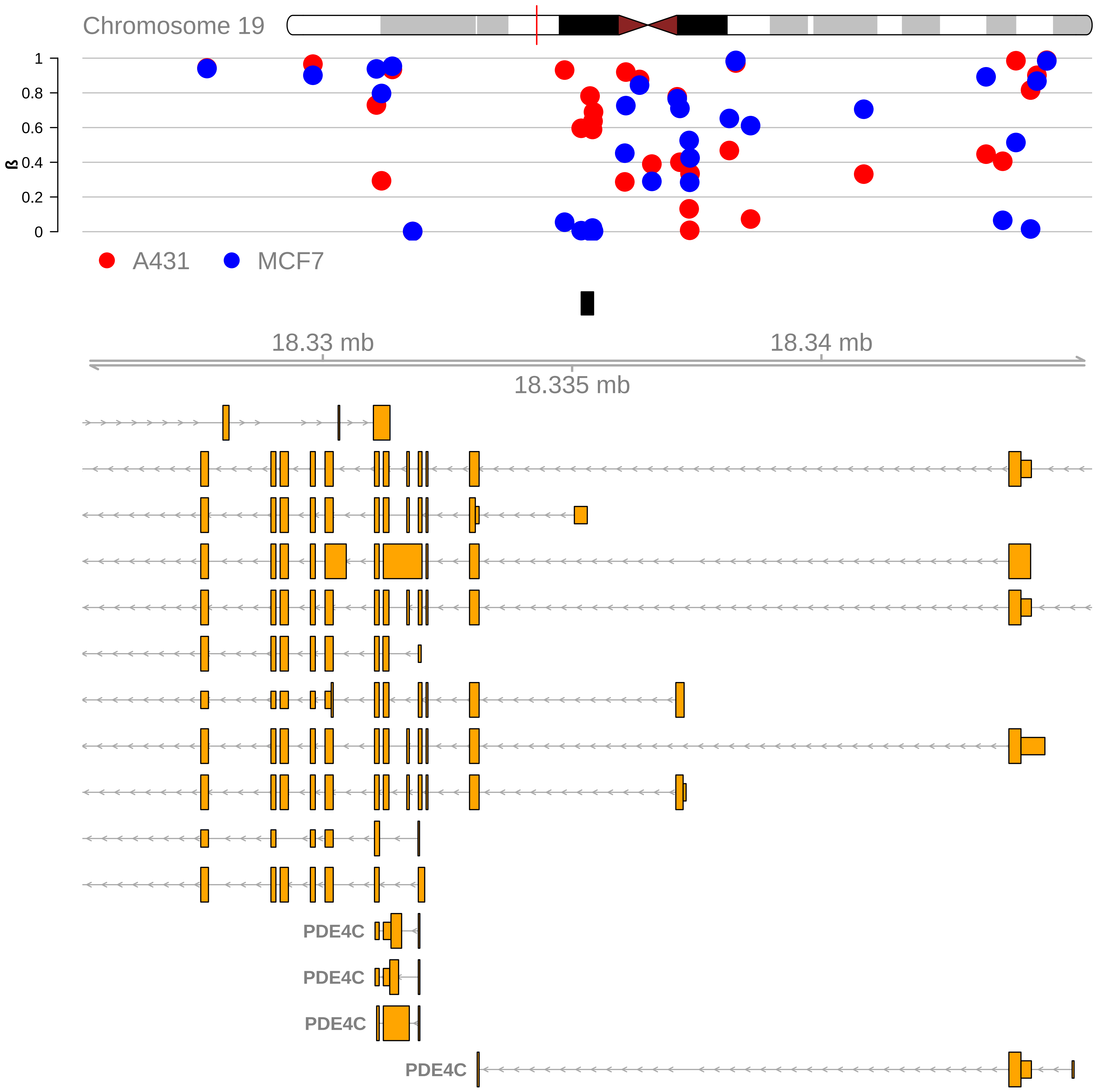

Chromosome 19
1
0.8
0.6
ß 0.4
0.2
0
A431
MCF7
18.33 mb
18.34 mb
18.335 mb
PDE4C
PDE4C
PDE4C
PDE4C
